# Portable Mass Spectrometry for In-Field Real-Time Water Pollution Monitoring: Validation and Pilot Study in Danube–Tisa–Danube Irrigation System

**DOI:** 10.3390/molecules31183164

**Published:** 2026-09-08

**Authors:** Djordje Vujić, Milena Aleksić, Daria Ilić, Boris Brkić

**Affiliations:** BioSense Institute, University of Novi Sad, Dr Zorana Đinđića 1, 21000 Novi Sad, Serbia; milena.aleksic@biosense.rs (M.A.); daria.ilic@biosense.rs (D.I.)

**Keywords:** membrane inlet mass spectrometer, volatile organic compounds, water pollution monitoring, hydrocarbons

## Abstract

Continuous, real-time monitoring of volatile organic compounds (VOCs) in surface water is critical for environmental protection, yet conventional laboratory Gas Chromatography–Mass Spectrometry (GC-MS) suffers from analyte loss during sample transport. This study field-validates a portable Membrane Inlet Mass Spectrometry (MIMS) system for direct, on-site monitoring of seven target VOCs (benzene, toluene, xylenes, chlorobenzene, 1,2-dichloroethane, trichloroethylene, and tetrachloroethylene). Laboratory validation established limits of detection between 4 and 8 µg/L, linearity (R^2^ > 0.98), and acceptable precision and accuracy per AOAC guidelines, benchmarked against headspace GC-MS. In-field testing at 36 locations across the Danube–Tisa–Danube (DTD) irrigation canal demonstrated system robustness. Baseline canal samples remained below the detection limits, but real-time MIMS successfully identified localized benzene and toluene contamination near a gasoline station. On-site MIMS detected higher VOC concentrations than delayed laboratory GC-MS, demonstrating its key advantage in preventing sampling volatilization losses. Portable MIMS proves to be a powerful, rapid screening tool for continuous aquatic environmental monitoring.

## 1. Introduction

The contamination of surface and groundwater resources by organic micropollutants—primarily volatile organic compounds such as aromatic hydrocarbons (BTEX) and chlorinated solvents, alongside agrochemicals and various other pollutants—represents a critical global environmental concern. These hazardous substances enter aquatic systems, including irrigation canals, drainage streams, and groundwater matrices, through industrial activities and accidental spillages [1,2]. Moreover, catastrophic natural events, such as major floods, can further exacerbate the situation by flushing industrial sites and permanently depositing pollutants into river overbank sediments and floodplains [3]. In surface water bodies used for agricultural irrigation, such as the complex hydro-system Danube–Tisa–Danube (DTD), these toxic compounds pose a dual threat. On one hand, they can enter the agri-food chain through soil-to-crop transfer, potentially endangering food safety from “farm-to-fork”. On the other hand, chronic exposure to VOCs through contaminated agricultural and environmental matrices is associated with adverse effects on the human immune and nervous systems, as well as vital internal organs. Because of the high volatility, toxicity, and ecological risks associated with these substances, developing highly sensitive laboratory analytical methods, real-time field monitoring networks, and advanced computational modeling tools is paramount for comprehensive environmental risk assessments [2,4]. The environmental significance of VOC monitoring is further reinforced by increasingly stringent international regulatory frameworks governing surface-water quality. Within the European Union, the Water Framework Directive (2000/60/EC) [5] and the Environmental Quality Standards Directive (2008/105/EC, amended by 2013/39/EU) [6] establish Environmental Quality Standards (EQSs), defining both annual average (AA-EQS) and maximum allowable concentrations (MAC-EQS) for hazardous organic contaminants. For example, the annual average environmental quality standard for benzene in surface waters is set at 10 μg L^−1^, while chlorinated solvents such as 1,2-dichloroethane and chloroform are subject to similarly strict concentration limits. Comparable regulatory requirements are enforced by the United States Environmental Protection Agency (US EPA) through the National Recommended Water Quality Criteria under the Clean Water Act [7], where VOCs, including benzene, trichloroethylene (TCE), and tetrachloroethylene (PCE), are regulated at low μg L^−1^ levels because of their ecological and human health risks. In Serbia, the Regulation on Limit Values of Pollutants in Surface and Ground Waters and Sediments [8], harmonized with the European legislative framework, prescribes equivalent threshold values for priority pollutants in surface waters intended for agricultural and environmental use. Compliance with these regulations requires reliable, rapid, and cost-effective analytical methods that support continuous environmental surveillance and early detection of contamination events.

Gas Chromatography–Mass Spectrometry (GC-MS) is the primary analytical technique for identifying and quantifying pollutants (e.g., organic pollutants, including volatile chlorinated hydrocarbons, BTEX, persistent organic pollutants—POPs, pesticide residues, etc.) in water networks [1,2,4,9]. Utilizing full mass spectra scans and contrasting data with extensive mass spectral libraries enables the precise, unambiguous identification of individual target compounds as opposed to relying on non-specific group parameters [2,4].

For the determination of target analytes such as benzene, toluene, xylenes, chlorobenzene, 1,2-dichloroethane, trichloroethylene, and tetrachloroethylene, several conventional extraction procedures coupled to mass spectrometry have been established, each possessing distinct operational trade-offs [10,11]. Given their high volatility and low concentration, the main analytical challenge in VOC detection is sample preparation. Water samples generally require extraction techniques such as Solid-Phase Extraction (SPE) or liquid–liquid extraction prior to analysis [9]. However, for volatile chlorinated hydrocarbons and BTEX in aqueous matrices, the purge-and-trap (P&T) technique has proven superior to classical extraction methods and offers outstanding sensitivity and low detection limits for trace-level concentrations, but it is limited by complex, expensive, and specialized experimental setups [10,12]. Headspace (HS) and headspace solid-phase microextraction (HS-SPME) are highly efficient, eco-friendly methods that avoid using hazardous organic solvents and are commonly used to extract BTEX and petroleum residues from complex solid and liquid samples. However, they often show poor repeatability across different samples. Static headspace, in particular, has low sensitivity because of limited injection volumes and the potential for thermal degradation [11,13,14].

Named advances in sampling and sample preparation during conventional laboratory workflow are welcomed, and they surely improve the sensitivity and accuracy of the analytical process. However, transport and storage still result in substantial volatile analyte losses. Additionally, conventional GC-MS analysis provides insight at specific time points due to discrete sampling. Thus, continuous monitoring of VOC concentration changes in emergent situations (e.g., oil spillages) is impossible.

Currently, to achieve rapid detection and continuous observation of water pollution, environmental monitoring frameworks are increasingly shifting toward automated sensor networks and field-deployable systems. This approach requires analytical instruments that can support rapid, sensitive, and real-time quantification of VOCs directly in the field [15]. Although conventional laboratory-based techniques offer superior accuracy and sensitivity, they cannot provide fast, real-time, on-site analysis. For immediate field screening of volatilized compounds, portable multi-gas sensors are routinely deployed; however, these field instruments can be highly susceptible to cross-sensitivities, necessitating fixed laboratory confirmation to rule out false positives [16]. On the other hand, mass spectrometry-based techniques are well known to be very sensitive, selective, accurate, and precise. The fundamental mechanism of MIMS relies on pervaporation through a selective membrane (usually silicone-based), which allows target VOCs to pass directly into the mass spectrometer’s ion source, entirely bypassing sample preparation [17,18]. Additionally, it provides both discrete analysis at specific time points, and continuous monitoring, with low power consumption. While this system has already been validated in the laboratory for BTX analysis [19], this study expands the target list to a wider array of volatile water pollutants and validates the system for the mixture of selected compounds in the laboratory and in the field, at 36 locations across the Danube–Tisa–Danube (DTD) irrigation canal in Serbia. In parallel, a similar MIMS system developed by the same research group has been successfully deployed for the on-site detection of volatile pollutants in the open sea [20]. The results obtained from both studies demonstrate the MIMS instrument’s potential as a very promising screening instrument for environmental monitoring.

## 2. Results

### 2.1. Laboratory Validation of Portable Mass Spectrometer

The MIMS system capability testing included examination of several parameters that are usually examined during the method validation process: selectivity, sensitivity (limits of detection and quantification), linearity, precision and accuracy. To validate the performance of the analytical method for target volatile organic compounds, specific criteria had to be met across five key parameters. First, selective permeation required the presence of characteristic mass fragments in scanned mass spectra for target VOC standards, as well as unit resolution for the selected *m*/*z* fragments used in monitoring and quantification. Sufficient sensitivity was established by achieving limits of detection relevant to legislative standards in the low parts-per-billion (ppb) range, maintaining a signal-to-noise ratio (S/N) greater than three. Additionally, a linear calibration range had to yield a coefficient of determination (R^2^) greater than 0.95 across the optimal concentration range. Finally, method validation involved determining both the precision and accuracy of the newly developed analytical approach.

The listed parameters were assessed according to the following plan:

- Selectivity examination included scanning the gas phase of pure chemicals for all target VOCs to obtain their mass fragmentation patterns using the MIMS system with an electron impact (EI) ion source and comparing them to the NIST database. Additionally, the mixture of all selected VOCs in the water matrix was scanned in order to confirm unit resolution.
- Sensitivity was examined by consecutive analyses of 12 water samples spiked at low ppb concentration levels to determine the limits of detection (LODs) and quantification (LOQs) for each VOC.
- The linearity of the method was assessed by analyzing water samples spiked at six concentration levels and determining the coefficient of determination (R^2^).
- Precision was examined by analyzing 12 water samples spiked at a concentration level of 50 ppb (µg/L)—six series with two samples per each day. From the results obtained, several parameters were calculated:
- Sw (%)—intra-serial standard deviation—expressing the repeatability of the method.
- RSD (%)—relative standard deviation—expressing the intra-laboratory repeatability of the method.
- Accuracy was examined by calculating the recovery (%) values for the 12 water samples spiked at the concentration level of 50 ppb (µg/L).

In addition to the evaluation of the listed validation parameters using the newly developed MIMS sensor and method, a parallel analysis using the conventional GC-MS method was performed for benchmarking purposes. Six water samples were spiked at a concentration level of 50 ppb for each target VOC and were analyzed to compare precision and accuracy between the two techniques.

#### 2.1.1. Selectivity Examination

Ethylbenzene and xylenes share the characteristic fragments *m*/*z* 91 and 106, and the literature indicates that xylenes are typically more abundant in water matrices. Ethylbenzene was excluded from the target analyte list. As a result, only xylenes will be monitored in further experiments, with the caveat that the reported xylene signals may include a minor contribution from ethylbenzene.

**Figure 1:**
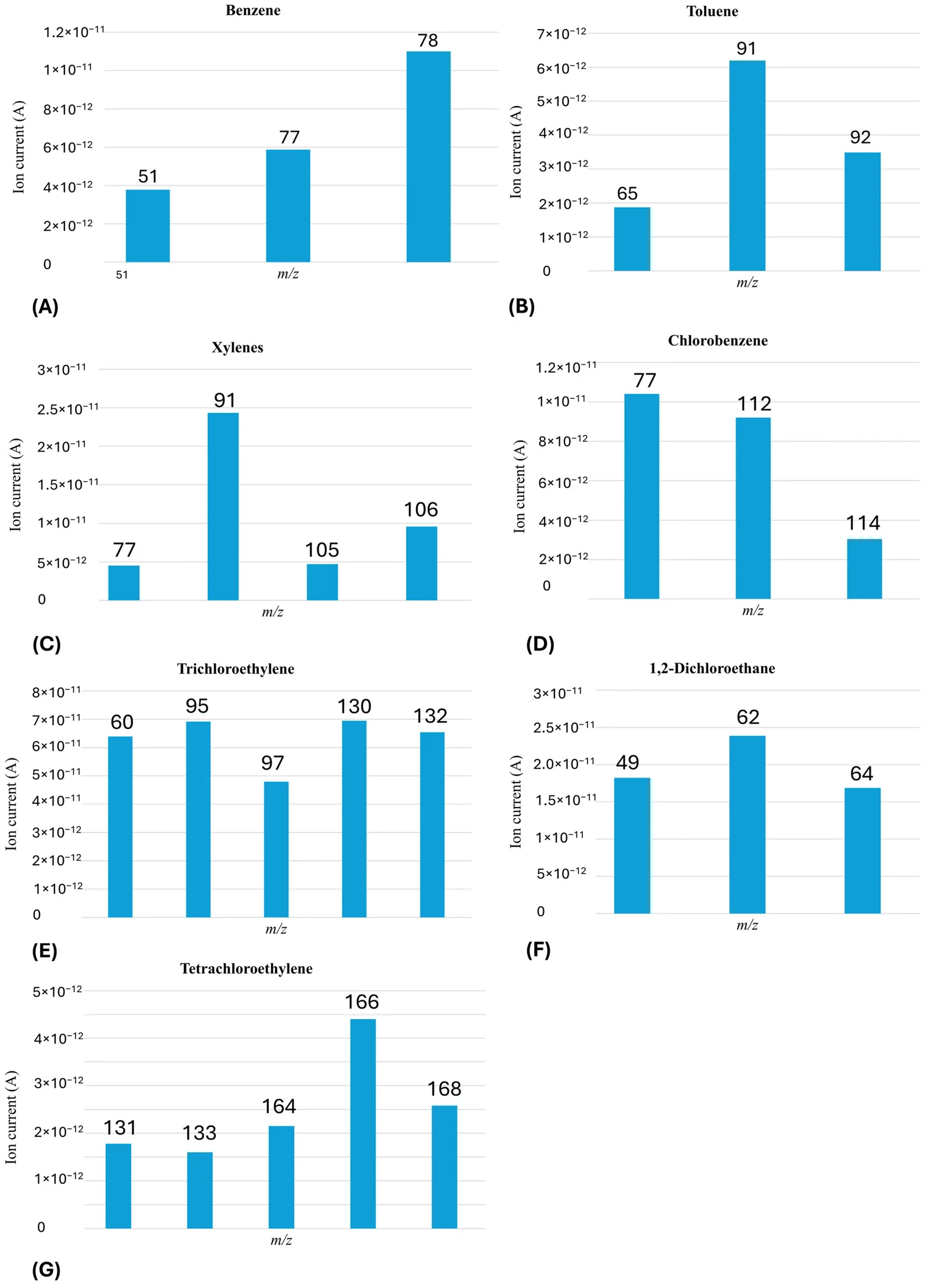
The dominant ions and their intensities obtained by MIMS for target VOCs, (**A**) Benzene, (**B**) Toluene, (**C**) Xylenes, (**D**) Chlorobenzene, (**E**) Trichloroethylene, (**F**) 1,2-dichloroethane, (**G**) Tetrachloroethylene.

The first step in selectivity confirmation was the examination of characteristic mass fragments in the scanned spectra using the MIMS sensor. Figure 1 shows the scanned spectra for benzene, toluene, xylenes, chlorobenzene, 1,2-dichlorethane, trichloroethylene and tetrachloroethylene. The acquired mass spectra were compared to the NIST database, and all evaluated compounds produced the expected mass fragments and corresponding fragment ratios.

Considering the obtained mass spectra, a single *m*/*z* fragment was selected for the following quantification for each of the target compounds. For this purpose, the most abundant fragment that was unique and did not overlap with fragments from any other VOC (Table 1) on the list was chosen.

**Figure 2:**
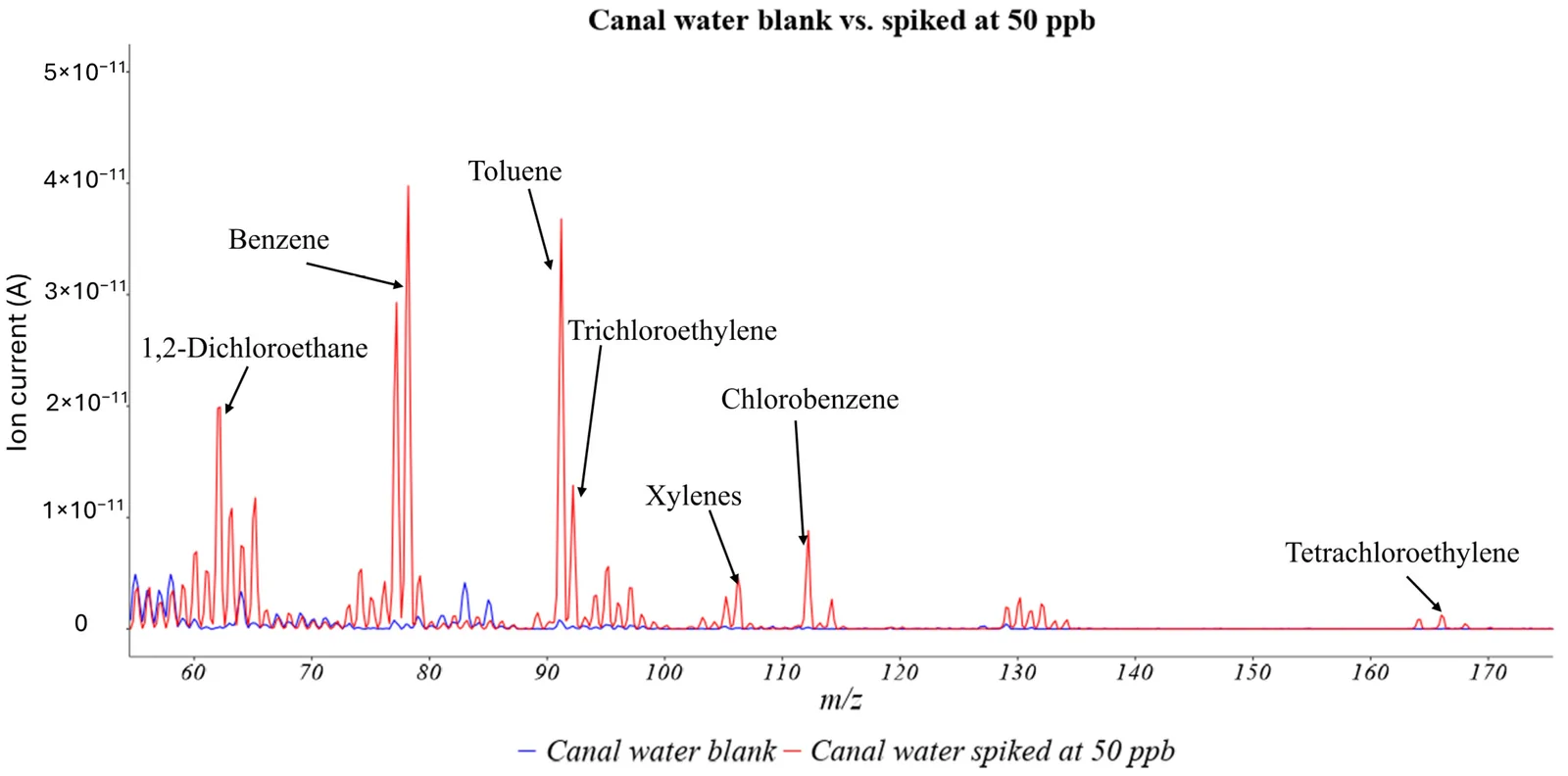
Scanned spectra of water sample spiked with mix of VOCs at concentration level of 50 ug/L (ppb) with marked selected *m*/*z* values selected for each VOC monitored.

The second step in selectivity evaluation was the examination of the resolution for each of the selected mass fragments. For this purpose, full spectral scans of blank and spiked canal water were compared (Figure 2). The unit resolution was confirmed for all examined target VOCs. The obtained results indicated that selective analysis for target compounds can be performed using the new MIMS sensor. It should be noted that for targeted quantification, the selected ion monitoring (SIM) mode was used. Operating in SIM mode allowed better sensitivity and signal scaling for each diagnostic *m*/*z* channel separately. This prevents intense fragments from masking lower-abundance ions and ensures reliable spectral interpretability and quantitative accuracy across the target dynamic range. The raw data used to generate Figure 1 is provided in the Supplementary Data as DataSet S1.

#### 2.1.2. Sensitivity Examination

The sensitivity of the new MIMS sensor and the corresponding analytical method was described via the limits of detection (LODs) and limits of quantification (LOQs) for each VOC. For LOD and LOQ determination, 12 consecutive measurements of water samples spiked at low concentrations for each VOC were conducted. Prior to formal LOD and LOQ calculations, preliminary range-finding experiments were conducted to establish the lowest concentration for each compound that maintained a signal-to-noise (S/N) ratio of ≥3. Following these preliminary experiments, the lowest calibration level for each VOC was used: 5 ppb for benzene, toluene, chlorobenzene, 1,2-dichloroethane, trichloroethylene and tetrachloroethylene and 15 ppb for xylenes.

The obtained signals were used for LOD and LOQ calculations. The following formulas were used. For LODs, we used the formula LOD = 3 × stddev ÷ slope, while for LOQs, we used the formula LOQ = 10 × stddev ÷ slope. LOD and LOQ values are summarized in Table 2. Data used to generate Table 2 were presented in the Supplementary Data as DataSet S2.

Given these limits of detection and quantification, the current configuration is highly suitable for general screening purposes. However, it may not satisfy the strictest drinking water regulations. Future efforts to reduce the LODs and LOQs could involve adopting hollow fiber membrane geometries or condensed phase membrane extraction, which have been successfully employed by other researchers to enhance VOC sensitivity [21,22]. While such hardware additions successfully reduce detection limits, they also compromise the simplicity of the system. Consequently, a practical trade-off must be established for each implementation based on the primary purpose of the device.

#### 2.1.3. Linearity Examination

**Figure 3:**
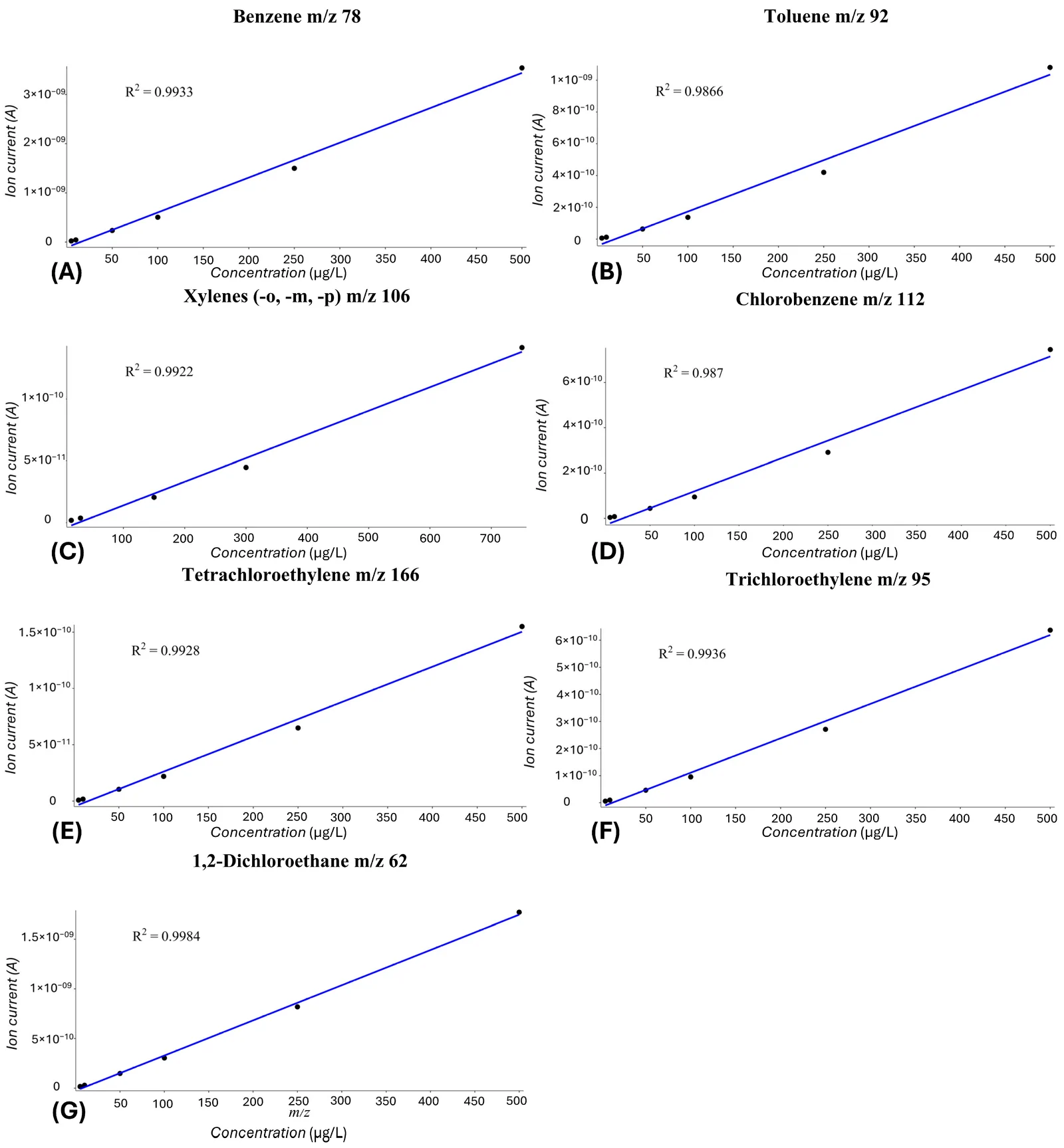
Calibration curves for target VOCs: (**A**) benzene, (**B**) toluene, (**C**) xylenes (-o, -m, -p), (**D**) chlorobenzene, (**E**) 1,2-dichlorothane, (**F**) trichloroethylene, and (**G**) tetrachloroethylene.

The linearity of the new analytical method using the MIMS sensor was determined using an in-house developed application for data processing, after construction of the calibration curves for each target VOC (Figure 3) in concentration ranges (Table 3). Raw data used to generate Table 3 were provided in the Supplementary Data as DataSet S3, while the calculations were provided in DataSet S2.

#### 2.1.4. Precision and Accuracy Examination

To determine the precision and accuracy of the new MIMS sensor and method, 12 water samples were spiked at a concentration level of 50 µg/L. The samples were prepared in six series of two samples. In the same way, a separate set of samples was prepared and analyzed by the conventional HS-GC-MS technique, which is the “gold standard” in VOC analysis and serves to benchmark the new technique. The analytes were quantified using corresponding calibration curves constructed separately on the MIMS and GC-MS instruments, as can be seen in Table 4. Calculations and data for Table 4 were provided in the DataSet S2 of the Supplementary Materials.

The precision of the MIMS and HS-GC-MS methods was defined through the repeatability and reproducibility of the method. The intra-serial standard deviation (Sw, %) is expressed as a measure of repeatability, while the relative standard deviation (RSD, %) within all analyzed samples is used for intra-laboratory reproducibility determination. Table 5 shows the obtained results for precision determination for the MIMS and HS-GC-MS techniques separately.

The accuracy of the MIMS and HS-GC-MS methods is expressed as recovery (%), which is calculated as:

$$Recovery(\%)=\frac{quantified\;value\;in\frac{ug}{L}}{spiked\;value\;in\frac{ug}{L}}\times 100.$$

The raw data used to generate Table 5 were presented in the Supplementary Data as DataSet S2.

In accordance with the set criteria according to AOAC [23], both the MIMS and HS-GC-MS methods provided acceptable precision and accuracy (Table 5). Notably, the recoveries for toluene and xylenes were lower than those of the other compounds. This reduction can likely be attributed to their larger molecular volume, lower vapor pressures, and increased membrane affinity resulting from their strongly non-polar nature.

Additionally, six water samples were spiked at the concentration level of 50 µg/L and analyzed in parallel by MIMS and HS-GC-MS, and the results are presented in Table 6.

The discrepancy between the results obtained using the GC-MS and MIMS techniques was evaluated using the combined relative standard deviations for each analyte, derived according to Eurachem [24] uncertainty propagation principles and calculated by the formula: ${RSD}_{comb}=\sqrt{{RSD}_{MIMS}^{2}+{RSD}_{GCMS}^{2}}$, using the RSD values obtained during the validation of both techniques (Table 5). The critical difference at the 95% confidence level was defined as *CD*_0.95_ = $1.96\times \sqrt{n}\times RSDcomb$, representing the maximum acceptable relative deviation between single results from both techniques. Accordingly, the upper limit for the relative standard deviation of a measurement pair (n=2) was established as *RSD_limit_ = CD*_0.95_/$\sqrt{n}$ *=* 1.96 × RSDcomb. These values are presented in Table 6 as the acceptance criteria for evaluating the consistency of results across different techniques. The dataset used to generate Table 6 was presented in DataSet S2.

Parallel analysis of the water samples spiked at 50 µg/L yielded relative standard deviations (RSDs) between the two techniques that were well below the predefined evaluation criterion for all samples and analytes. The raw data used to generate Figure 3 were presented in the Supplementary Data as DataSet S3.

### 2.2. In-Field Tests

The validated MIMS system and corresponding analytical method were employed for direct and real-time analysis of target VOCs (Table 7) in a real environment along a section of the Danube–Tisa–Danube irrigation system.

**Figure 4:**
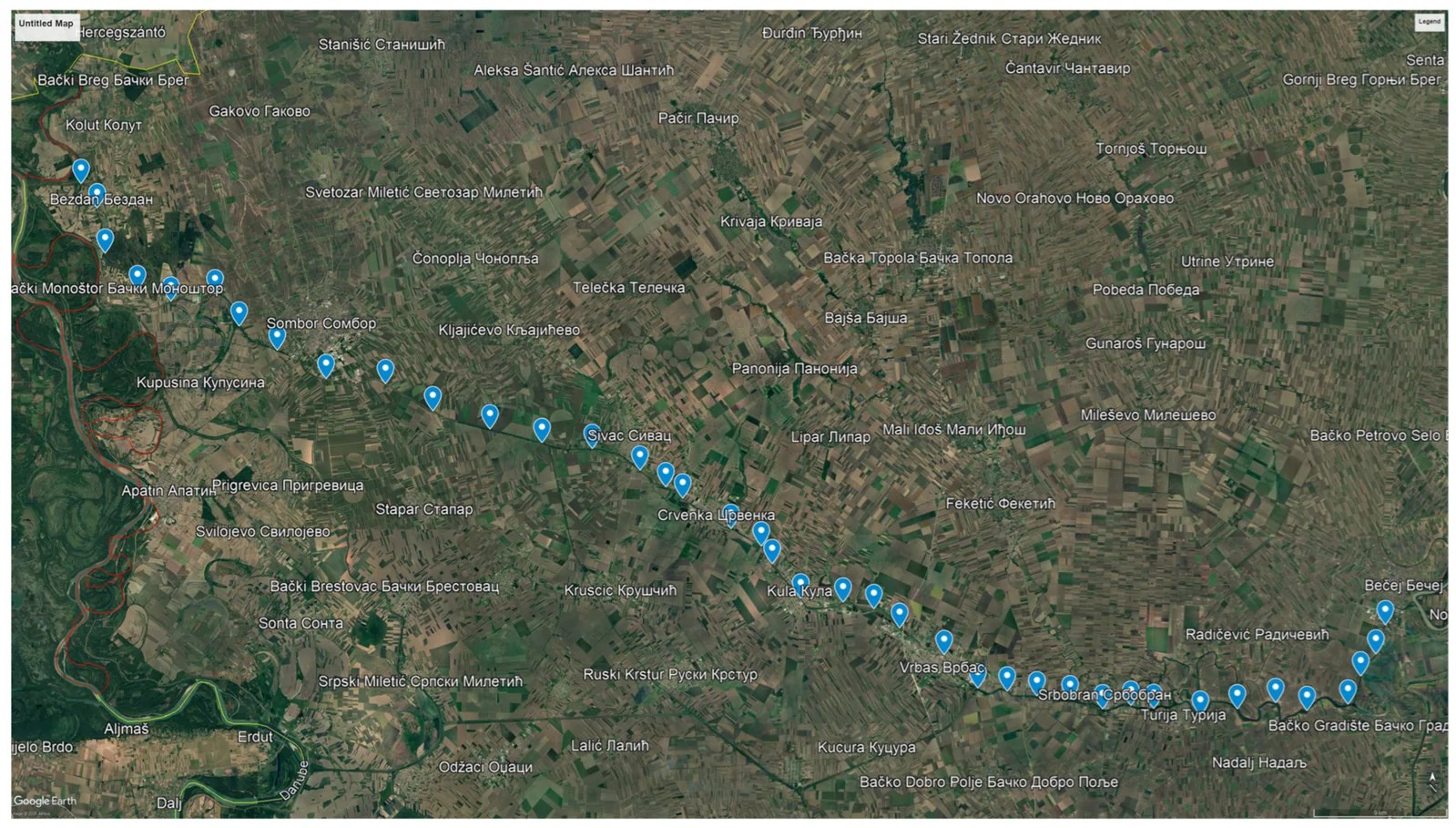
Map of selected sampling locations (Base map generated using Google Earth Pro (v7.3.7.1155); satellite imagery © 2026 Airbus, map data © 2026 Google). Blue pins indicate exact sampling sites.

In-field validation of the MIMS system was conducted during the period of April–May 2026. In total, 40 locations (Figure 4) were selected for sampling; however, four locations were not accessible. Therefore, the total number of locations examined in this study was 36, analyzed by two analytical techniques—the newly developed portable MIMS system and GC-MS, in laboratory conditions. The MIMS system was used in real-time on-site analysis, while GC-MS samples were collected in triplicate and analyzed on the same day in the laboratory. Fresh calibration standards were prepared prior to every analysis for each technique separately.

#### 2.2.1. MIMS Results

An in-house application was created for VOC quantification. It enables processing of the scan and SIM (selected ion monitoring) raw files generated by MIMS. The first step in the current research data processing includes the creation of calibration curves based on the MIMS-generated SIM (selected ion monitoring) files for each analyte, at different concentration levels. Once calibration curves are set, sample files are plotted against the calibration curves and quantified ppb values are obtained. VOC concentrations are provided in .csv format.

During in-field validation of the portable VOC sensor, sampling was conducted at 36 locations along the DTD canal, and none of the examined VOCs were present in concentration above the LODs obtained during method validation. The results are presented in Table 8 with sample IDs for 36 locations only. The raw data used to generate Table 8 were presented in the Supplementary Data as DataSet S4.

**Figure 5:**
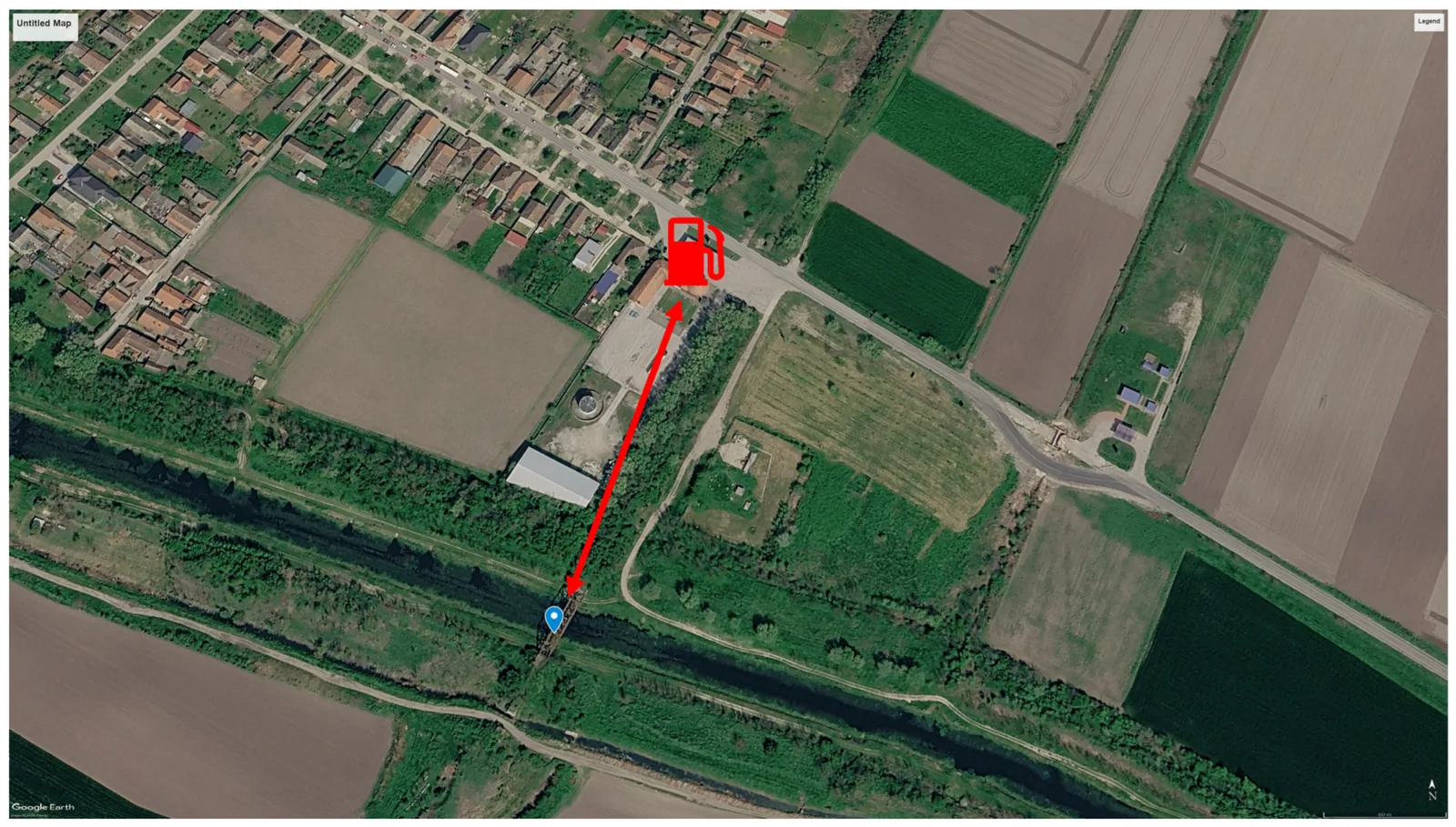
Sampling location near gasoline station (Base map generated using Google Earth Pro (v7.3.7.1155); satellite imagery © 2026 Airbus, map data © 2026 Google). The red arrow indicates the distance of the sampling site to the gas station.

Even though there are no quantified values for the examined VOCs throughout the irrigation system, at one location, increased signals for benzene and toluene were detected by MIMS. This location is 200 m from the gasoline station (Figure 5). Thus, the obtained results are logical, as ambient concentrations of benzene and toluene primarily originate from anthropogenic sources, including motor vehicle exhaust, fossil fuel combustion (coal and oil), and fugitive evaporative emissions from gasoline service stations [26,27].

**Figure 6:**
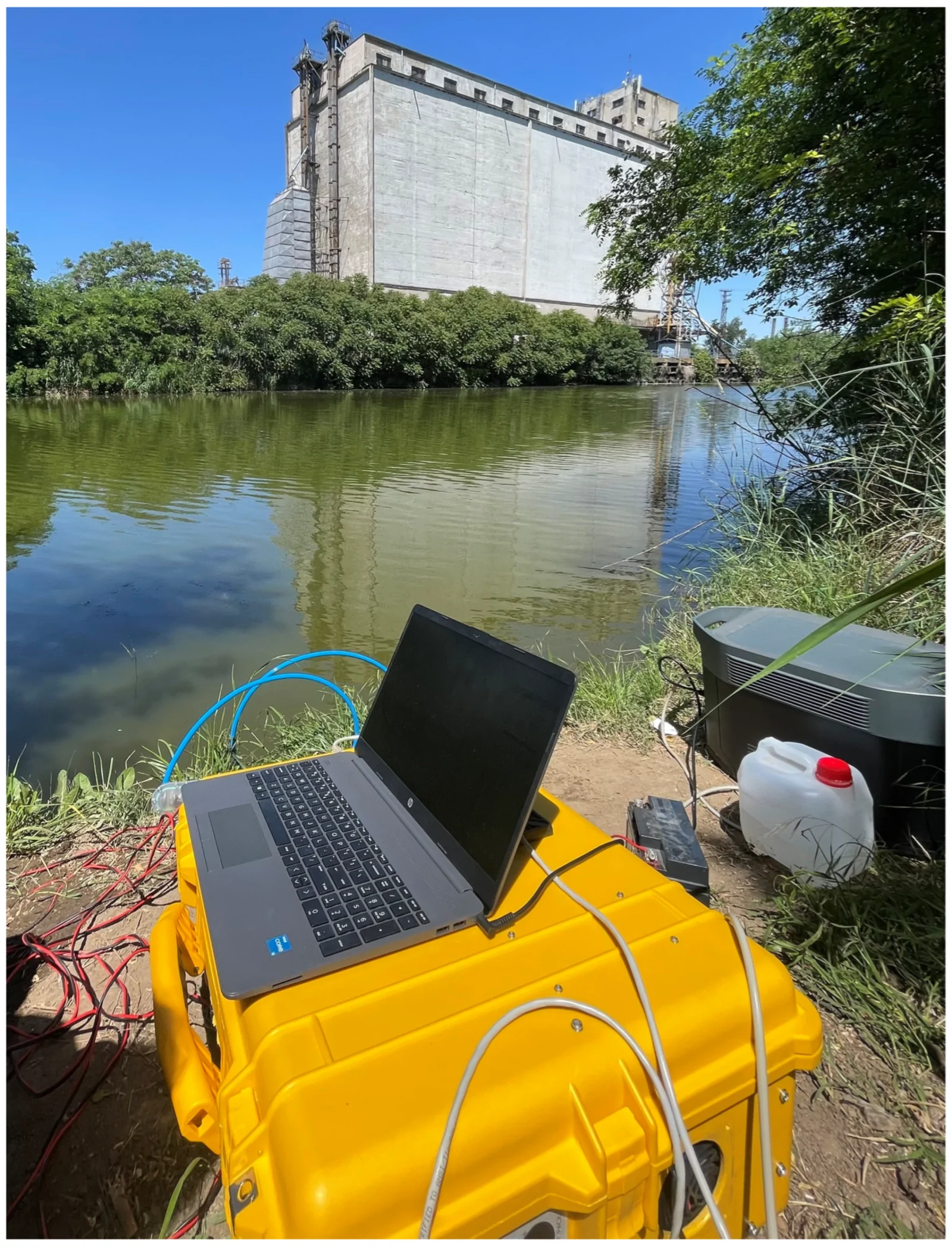
Deployment configuration of the MIMS for VOC monitoring.

Figure 6 illustrates the active in situ deployment of the MIMS sensor. The external battery for powering the MIMS can be seen, as well as the battery for powering the pump for sample pumping.

#### 2.2.2. GC-MS Results

The conventional GC-MS technique confirmed the results obtained by the MIMS sensor. No examined VOCs were above the limits of quantification or limits of detection for the MIMS sensor. However, some of the VOCs were detected by GC-MS in some samples. Qualitative presence was confirmed by the NIST database incorporated into the GC-MS results’ processing software (Agilent^®^ MassHunter Quantitative Analysis, version 10.2) and by comparing the mass spectra of VOCs detected in the samples with the mass spectra of calibration standards for the corresponding VOCs. The results are presented as detected (marked as “o” in Table 9) and non-detected (marked as “x”), and the sample locations numbered L7, L20, L23 and L37 were inaccessible at the moment of sampling. VOC levels are not quantified, as detected values were outside the performed calibration range. These results serve as an indication of the presence of the examined VOCs.

As can be seen, toluene and tetrachloroethylene were detected at all examined locations. These are followed by chlorobenzene, which was detected at 26 locations, xylenes, which were present at nine locations, trichloroethylene, which was present at six locations, and benzene, which was present at two locations. 1,2-dichloroethane was not detected at any location.

The dataset used to generate Table 9 are provided in the Supplementary Data as DataSet S5.

The ubiquity of toluene and tetrachloroethylene (PCE) across all monitored sites is a direct consequence of their widespread anthropogenic application and environmental persistence. Toluene typically enters the canal network via urban stormwater runoff and industrial effluents, whereas PCE—primarily utilized as a solvent in dry cleaning and metal degreasing—exhibits high resistance to biodegradation in surface aquatic environments. The high detection frequency of chlorobenzene (at 26 locations) aligns with the regional profile of the Danube–Tisza–Danube canal basin, as this compound serves as a critical intermediate in pesticide and dye synthesis, directly reflecting the intensive agro-industrial activities characterizing the Vojvodina region. Conversely, xylenes (nine locations) and trichloroethylene (TCE) (six locations) were detected less frequently. Although xylenes share similar pyrogenic and industrial sources with toluene, they undergo substantially faster photolytic and microbial degradation in open lotic systems, while TCE occurrences are typically confined to localized, point-source discharges from metal processing facilities. The sparse detection of benzene at only two locations is justified by its physicochemical properties. Despite its prevalence in petroleum derivatives, its exceptionally high vapor pressure drives rapid volatilization from surface waters into the atmosphere, compounded by strict regulatory restrictions implemented due to its high toxicity. Finally, the complete absence of 1,2-dichloroethane was expected, as its historical use as an anti-knock additive in leaded gasoline has been entirely phased out and its contemporary application in PVC synthesis is confined to strictly closed-loop industrial processes, preventing its adventitious release into open hydro systems.

#### 2.2.3. MIMS vs. HS-GC-MS Results

Given that we detected only benzene and toluene at only one location using the MIMS sensor, we can make comparisons of the results only for these compounds between the two techniques. At this specific location, the quantified values obtained by MIMS were 2.87 ppb for benzene and 3.04 ppb for toluene, respectively. For the same location, the quantitative values obtained by GC-MS were 0.21 ppb for benzene and 0.55 ppb for toluene. It must be noted that these values are just indicative, as they were outside the calibration range. It can be observed that the MIMS results were higher in value than the results obtained by delayed analysis by GC-MS. As we stated, this was the location near the gasoline station, and given that these VOCs evaporate very fast, it is logical that the on-site technique could detect a greater amount compared to the delayed analysis by GC-MS. Even though it is only one location, this is a very promising outcome and proof that on-site monitoring solutions are highly needed. On the other hand, at other locations, when examined pollutants were present, but not in sufficient amounts (below the MIMS sensor’s limits of detection), these were still detectable by the conventional technique. Therefore, these results are real confirmation that both approaches are needed for holistic environmental monitoring. Further testing and statistical analysis are needed to draw a definite conclusion.

The results of this research, which consistently found volatile organic compound concentrations below the limits of quantification, generally align with the regional water quality data presented in a broader monitoring report. For major monitored waterways, including the Danube, Tisa, Timok, and Mlava rivers, concentrations of key target compounds such as benzene, 1,2-dichloroethane, trichloroethylene, and tetrachloroethylene were overwhelmingly reported below the detection limit of <0.005 µg/L. However, while this regional report [28] serves as the closest available baseline, direct comparability is limited. Our sampling campaign was conducted exclusively within the Danube–Tisa–Danube canal network, which faces distinctly different anthropogenic pressures than the main river courses. Notably, a significant proportion of the region’s heavy industry is situated directly along the DTD canal rather than the Danube or Tisa rivers, theoretically elevating the localized risk of industrial discharge. Even with this higher risk of contamination, VOC levels remained consistently below the LOQs, indicating a very low baseline of VOCs throughout the region’s waters. This underscores the importance of using a highly sensitive analytical procedure like ours for monitoring industrialized aquatic systems.

## 3. Materials and Methods

### 3.1. Chemicals and Reagents

All the VOCs [29,30,31,32,33,34,35] used in this study were acquired from Sigma-Aldrich Chemie GmbH (Taufkirchen, Germany). Membranes used in the membrane inlet were acquired from Technical Products Inc., Georgia (Buford, GA, USA). To generate calibration curves, aqueous standards were freshly prepared at the deployment site by spiking canal water with serially diluted VOC stock solutions in HPLC-grade ethanol. All preparations were handled in sealed glass vials to prevent volatilization losses prior to immediate, on-site mass spectrometer calibration.

### 3.2. Instrumentation—Membrane Inlet Mass Spectrometer

Mass spectra (*m*/*z* 1–300, SIM mode) were acquired on a PrismaPro QMG 250 M3 quadrupole mass spectrometer (Pfeiffer Vacuum GmbH, Asslar, Germany) using 70 eV electron ionization via an yttria-coated tungsten filament (2000 µA current) and a combined Faraday/electron multiplier detector. The unit was housed in a custom vacuum chamber (UltraHighVacuum, East Sussex, UK) maintained at 4 × 10^−6^ Torr using a Pfeiffer HiPace 80 turbomolecular pump, an MVP 030-3DC backing pump, and an MPT 200 cathode pressure gauge (all supplied by Pfeiffer Vacuum GmbH, Asslar, Germany). Sample delivery during field analysis was facilitated by a 12 V DC submersible bilge pump (Accredo Logistics, Novi Sad, Serbia). The system was controlled using PV MassSpec software (V.23.06).

**Figure 7:**
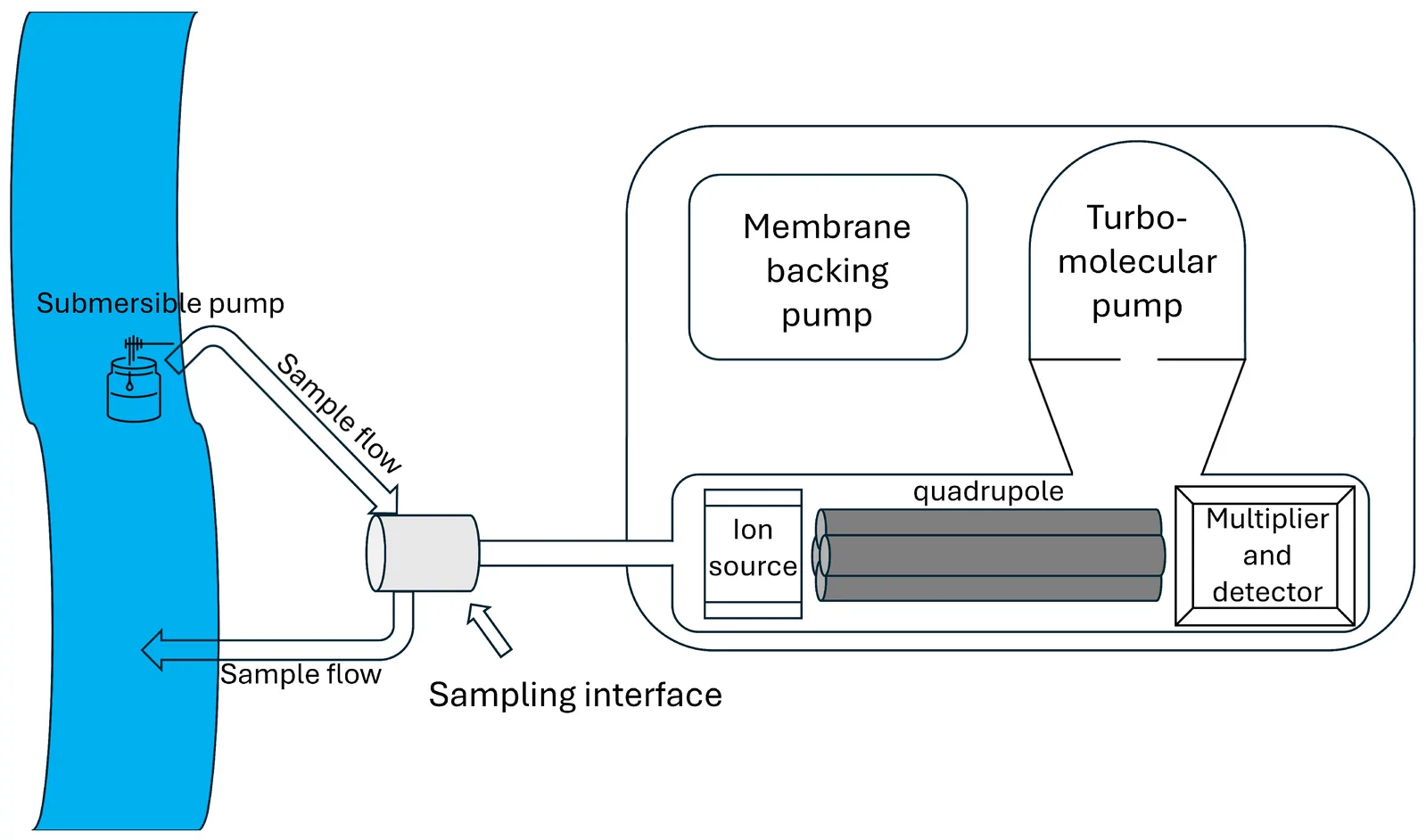
Schematic diagram of MIMS including sampling interface.

The membrane inlet, sampling interface, and internal component layout of the portable mass spectrometer were described in our previous study [20]. The MIMS system was packed in a protective case provided by Pelican (Torrance, CA, USA). The dimensions of the case were 60 × 50 × 23 cm (length × width × height), with the mass of the entire assembly being 29 kg. A schematic diagram of the MIMS, including the sampling interface, can be seen in Figure 7.

### 3.3. Instrumentation—GC-MS

Headspace sampling was executed using an automated PAL3 autosampler coupled to an Agilent 8890 Gas Chromatograph (GC) with an Agilent 5977B Mass Selective Detector (MSD) (Agilent Technologies, Santa Clara, CA, USA). Samples were equilibrated at 60 °C for 10 min under intermittent agitation (250 rpm; 10 s on, 20 s off). Headspace vapor (0.2 mL) was injected via a heated syringe (60 °C) into the GC inlet operated in split mode at 250 °C. Chromatographic separation was performed on an Agilent HP-5MS capillary column (Agilent Technologies, Santa Clara, CA, USA) (30 m × 0.25 mm i.d., 0.25 µm film thickness) using helium as the carrier gas at a constant flow rate of 1.0 mL/min. The oven temperature was initially held at 31 °C for 2.5 min, ramped at 7 °C/min to 150 °C, and maintained for 1.0 min, resulting in a total chromatographic run time of 20.5 min. Data acquisition and spectral processing were performed using Agilent MassHunter Workstation software (Version 10.2).

## 4. Conclusions

This study successfully validated and deployed a portable Membrane Inlet Mass Spectrometry (MIMS) system for direct, real-time on-site monitoring of target volatile organic compounds (VOCs)—including benzene, toluene, xylenes, chlorobenzene, 1,2-dichloroethane, trichloroethylene, and tetrachloroethylene—across 36 locations in the Danube–Tisa–Danube (DTD) irrigation canal network. Laboratory validation confirmed that the MIMS method fulfills the AOAC criteria for selectivity, sensitivity (limits of detection between 4 and 8 µg/L), linearity (R^2^ > 0.98), precision, and accuracy, showing strong analytical agreement with conventional headspace GC-MS. While the general baseline canal concentrations remained below the MIMS detection limits across the hydro-system, the sensor proved its field effectiveness near an active gasoline station, where on-site MIMS captured markedly higher concentrations of volatile benzene (2.87 µg/L) and toluene (3.04 µg/L) than delayed laboratory GC-MS analysis. This highlights the key capacity of MIMS to prevent volatile analyte losses caused by sample collection, handling, and storage. Ultimately, portable MIMS represents a robust, rapid, and solvent-free screening technology for continuous aquatic surveillance, demonstrating real-time field measurements. Together, fixed laboratory GC-MS and MIMS effectively complement each other to achieve holistic environmental risk assessment and early pollution detection. Future studies should incorporate multi-seasonal analyses, given that temporal variations in the water matrix can impact the sensitivity and selectivity of the analysis.

## Figures and Tables

**Table 1:** Mass (*m*/*z*) fragments for each target compound.

VOC	Benzene	Toluene	Xylenes	Chlorobenzene	1,2-Dichloroethane	Trichloroethylene	Tetrachloroethylene
*m*/*z*	78	92	106	112	62	95	166

**Table 2:** Results obtained for LOQs and LODs using MIMS sensor.

VOC	SD (%)	Slope	LOD (µg/L)	LOQ (µg/L)
Benzene	1.099 × 10^−11^	7.09 × 10^−12^	5	16
Toluene	3.135 × 10^−12^	2.16 × 10^−12^	5	15
Xylenes (-o, -m, -p)	4.822 × 10^−13^	1.91 × 10^−13^	8	25
Chlorobenzene	1.614 × 10^−12^	1.49 × 10^−12^	4	11
1,2-Dichloroethane	5.741 × 10^−12^	3.542 × 10^−12^	5	16
Trichloroethylene	2.519 × 10^−12^	1.27 × 10^−12^	7	20
Tetrachloroethylene	4.396 × 10^−13^	3.104 × 10^−13^	5	14

SD—standard deviation of 12 measurements.

**Table 3:** Linearity data obtained using the MIMS sensor.

VOC	Calibration Concentration Range (µg/L)	R^2^	Equation
Benzene	5–500	0.9933	Y = −1.057912 × 10^−10^ + 7.089013 × 10^−12^ × X
Toluene	5–500	0.9866	Y = −4.210876 × 10^−11^ + 2.154744 × 10^−12^ × X
xylenes (-o, -m, -p)	15–750	0.9922	Y = −5.0872 × 10^−12^ + 1.910303 × 10^−13^ × X
Chlorobenzene	5–500	0.9870	Y = −2.926351 × 10^−11^ + 1.489514 × 10^−12^ × X
1,2-Dichloroethane	5–500	0.9984	Y = −2.577647 × 10^−11^ + 3.541737 × 10^−12^ × X
Trichloroethylene	5–500	0.9936	Y = −1.624075 × 10^−11^ + 1.270488 × 10^−12^ × X
Tetrachloroethylene	5–500	0.9928	Y = −4.885442 × 10^−12^ + 3.104232 × 10^−13^ × X

Calibration curves and linearity parameters are represented in Figure 3.

**Table 4:** Quantification results using MIMS and HS-GC-MS for target VOCs: (**a**) benzene, (**b**) toluene, (**c**) xylenes (sum -o, -m, -p), (**d**) chlorobenzene, (**e**) 1,2-dichloroethane, (**f**) trichloroethylene, and (**g**) tetrachloroethylene.

(a)	MIMS (µg/L)	HS-GC-MS (µg/L)		MIMS (µg/L)	HS-GC-MS (µg/L)
1	41.03	47.99	2	39.27	47.67
3	38.14	47.11	4	35.20	46.75
5	35.19	41.28	6	33.45	48.55
7	36.97	46.38	8	32.51	46.26
9	31.32	46.19	10	32.16	40.43
11	33.15	44.54	12	31.88	45.54
(**b**)					
1	46.33	41.93	2	35.71	41.66
3	36.56	41.05	4	30.82	40.48
5	31.04	35.80	6	28.27	41.24
7	48.03	39.23	8	27.42	38.59
9	28.27	38.42	10	29.55	33.41
11	27.21	36.01	12	31.25	36.77
(**c**)					
1	47.64	114.55	2	35.98	113.61
3	34.63	112.05	4	29.70	109.76
5	23.87	97.70	6	44.05	111.07
7	24.77	105.14	8	26.11	103.66
9	23.87	102.05	10	30.60	88.35
11	25.22	94.50	12	27.46	96.81
(**d**)					
1	49.32	47.03	2	50.61	47.07
3	47.38	46.10	4	46.35	45.59
5	43.38	40.63	6	49.19	46.25
7	43.90	44.34	8	42.61	43.49
9	42.35	43.18	10	44.16	37.63
11	41.32	40.55	12	42.09	41.61
(**e**)					
1	45.86	55.70	2	43.07	56.14
3	42.49	54.96	4	40.28	55.82
5	41.46	48.80	6	39.70	57.35
7	41.02	56.08	8	38.52	56.02
9	37.35	45.86	10	37.20	50.05
11	38.38	54.80	12	38.52	56.01
(**f**)					
1	59.29	47.71	2	57.81	47.54
3	57.32	47.07	4	55.09	46.42
5	47.93	41.62	6	62.50	47.78
7	41.50	46.19	8	41.50	45.87
9	47.68	45.47	10	48.91	40.30
11	46.94	43.94	12	48.67	45.16
(**g**)					
1	68.06	49.27	2	55.11	49.05
3	48.48	48.84	4	47.81	48.25
5	47.48	44.04	6	71.14	49.47
7	40.18	47.87	8	40.18	47.76
9	36.86	47.73	10	37.19	42.34
11	33.21	45.91	12	34.21	47.44

**Table 5:** Precision and accuracy results obtained using newly developed MIMS sensor and HS-GC-MS instrument.

**VOC**	**MIMS Precision**					**MIMS Accuracy**
	**s_w_ (%)**	**s_x_ (%)**	**s_b_ (%)**	**s_tot_ (%)**	**RSD (%)**	**Recovery (%)**
Benzene	1.76	3.05	1.25	2.15	6.15	70
Toluene	7.05	5.07	2.07	7.34	22.01	67
Xylenes (sum -o, -m, -p)	7.19	6.22	2.54	7.62	24.46	62
Chlorobenzene	1.87	3.05	1.24	2.25	4.97	90
1,2-Dichloroethane	1.35	2.51	1.02	1.70	4.21	81
Trichloroethylene	4.32	6.47	2.64	5.07	9.88	103
Tetrachloroethylene	7.79	11.73	4.79	9.15	19.60	93
AOAC criteria	<21				<32	60–115
**VOC**	**HS-GC-MS Precision**					**HS-GC-MS Accuracy**
	**s_w_ (%)**	**s_x_ (%)**	**s_b_ (%)**	**s_tot_ (%)**	**RSD (%)**	**Recovery (%)**
Benzene	2.70	1.62	0.66	2.78	6.07	91
Toluene	2.16	2.32	0.95	2.36	6.10	77
o-Xylene	1.91	2.48	1.01	2.16	6.01	72
m-Xylene	1.81	2.56	1.04	2.09	6.27	67
p-Xylene	1.91	2.67	1.09	2.20	6.31	70
Chlorobenzene	2.32	2.59	1.06	2.55	5.84	87
1,2-Dichloroethane	3.02	1.41	0.57	3.07	5.61	110
Trichloroethylene	2.36	1.71	0.70	2.46	5.41	91
Tetrachloroethylene	2.26	1.49	0.61	2.34	4.95	95
AOAC criteria	<21				<32	60–115

**Table 6:** Results obtained in parallel by MIMS and HS-GC-MS analyses of the same spiked samples at 50 µg/L (ppb) for target VOCs: (**a**) benzene, (**b**) toluene, (**c**) xylenes (sum -o, -m, -p), (**d**) chlorobenzene, (**e**) 1,2-dichloroethane, (**f**) trichloroethylene, and (**g**) tetrachloroethylene.

(a)	MIMS (µg/L)	HS-GC-MS (µg/L)	RSD (%)
1	41.03	47.99	11.06
2	38.14	47.11	14.88
3	35.19	41.28	11.26
4	36.97	46.38	15.97
5	39.27	47.67	13.66
6	32.16	40.43	16,11
		RSD limit (%)	16.93
(**b**)			
1	43.36	50.33	10.52
2	34.43	48.41	23.86
3	35.50	48.44	21.81
4	30.19	49.96	34,89
5	30.82	49.48	32.86
6	40.38	49.47	14.31
		RSD limit (%)	44.76
(**c**)			
1	48.09	39.02	14.73
2	35.98	38.45	4.69
3	33.74	37.78	8.00
4	29.25	39.19	20.53
5	31.94	38.19	12.60
6	43.60	39.15	7.61
		RSD limit (%)	49.46
(**d**)			
1	56.03	63.76	9.13
2	50.35	61.27	13.84
3	50.61	60.77	12.91
4	51.87	63.00	13.71
5	58.99	62.37	3.95
6	61.19	62.61	1.62
		RSD limit (%)	15.03
(**e**)			
1	45.86	55.7	13.70
2	49.42	54.96	7.51
3	41.46	48.8	11.50
4	41.02	46.08	8.22
5	43.07	50.14	10.73
6	47.2	50.05	4.14
		RSD limit (%)	13.75
(**f**)			
1	61.27	56.75	5.41
2	61.27	54.53	8.22
3	58.80	54.63	5.19
4	58.06	56.59	1.80
5	52.62	55.81	4.16
6	53.36	56.05	3.48
		RSD limit (%)	22.08
(**g**)			
1	54.12	54.10	0.02
2	52.46	52.51	0.07
3	50.80	52.16	1.87
4	50.47	54.32	5.20
5	42.50	53.09	15.66
6	44.50	53.53	13.03
		RSD limit (%)	39.63

**Table 7:** The list of selected VOCs analyzed by MIMS [25].

VOC	CAS No.	Boiling Point (C)	Characteristic *m*/*z* According to NIST Database
Benzene	71-43-2	80.1	78, 77, 52, 51
Toluene	108-88-3	110.6	91, 65, 39
Xylenes (-m, -o, -p)	1330-20-7	138–144 (mixture)	91, 106, 77
1,2-Dichloroethane (1,2-DCE)	0107-06-02	83.5	62, 64, 27
Trichloroethylene (TCE)	79-01-6	87	95, 97, 130
Tetrachloroethylene (PCE)	127-18-4	121	166, 168, 131
Chlorobenzene	108-90-7	132	112, 114, 77

**Table 8:** MIMS results on examined VOCs in DTD canal water samples at 36 locations, in triplicate. B—benzene; T—toluene; X—xylenes (-o, -m, -p); 1,2-DCE—1,2-dichloroethane; TCE—trichloroethylene; PCE—tetrachloroethylene; CB—chlorobenzene.

VOCs Concentration (ppb)							
Sample ID	B	T	X	1,2-DCE	TCE	PCE	CB
L1	<5	<5	<8	<5	<7	<5	<4
L2	<5	<5	<8	<5	<7	<5	<4
L3	<5	<5	<8	<5	<7	<5	<4
L4	<5	<5	<8	<5	<7	<5	<4
L5	<5	<5	<8	<5	<7	<5	<4
L6	<5	<5	<8	<5	<7	<5	<4
L8	<5	<5	<8	<5	<7	<5	<4
L9	<5	<5	<8	<5	<7	<5	<4
L10	<5	<5	<8	<5	<7	<5	<4
L11	<5	<5	<8	<5	<7	<5	<4
L12	<5	<5	<8	<5	<7	<5	<4
L13	<5	<5	<8	<5	<7	<5	<4
L14	<5	<5	<8	<5	<7	<5	<4
L15	<5	<5	<8	<5	<7	<5	<4
L16	<5	<5	<8	<5	<7	<5	<4
L17	<5	<5	<8	<5	<7	<5	<4
L18	<5	<5	<8	<5	<7	<5	<4
L19	<5	<5	<8	<5	<7	<5	<4
L21	<5	<5	<8	<5	<7	<5	<4
L22	<5	<5	<8	<5	<7	<5	<4
L24	<5	<5	<8	<5	<7	<5	<4
L25	<5	<5	<8	<5	<7	<5	<4
L26	<5	<5	<8	<5	<7	<5	<4
L27	<5	<5	<8	<5	<7	<5	<4
L28	<5	<5	<8	<5	<7	<5	<4
L29	<5	<5	<8	<5	<7	<5	<4
L30	<5	<5	<8	<5	<7	<5	<4
L31	<5	<5	<8	<5	<7	<5	<4
L32	<5	<5	<8	<5	<7	<5	<4
L33	<5	<5	<8	<5	<7	<5	<4
L34	<5	<5	<8	<5	<7	<5	<4
L35	<5	<5	<8	<5	<7	<5	<4
L36	<5	<5	<8	<5	<7	<5	<4
L38	<5	<5	<8	<5	<7	<5	<4
L39	<5	<5	<8	<5	<7	<5	<4
L40	<5	<5	<8	<5	<7	<5	<4

**Table 9:** Heat map of GC-MS results on examined VOCs in DTD canal water samples at 36 locations, in triplicate. Green shading and circles ‘o’ denote sampling locations where VOC’s were found at LOD level. Red shading and ‘x’ indicate locations where VOC’s were bellow LOD while slashes ‘/’ represents locations that were inaccessible during the pilot sampling phase.

	B			T			X			CB			1,2-DCE			TCE			PCE		
ID	1	2	3	1	2	3	1	2	3	1	2	3	1	2	3	1	2	3	1	2	3
L1	o	o	o	o	o	o	x	x	x	o	o	o	x	x	x	x	x	x	o	o	o
L2	x	x	x	o	o	o	x	x	x	o	o	o	x	x	x	x	x	x	o	o	o
L3	x	x	x	o	o	o	x	x	x	o	o	o	x	x	x	x	x	x	o	o	o
L4	x	x	x	o	o	o	x	x	x	o	o	o	x	x	x	x	x	x	o	o	o
L5	x	x	x	o	o	o	x	x	x	o	o	o	x	x	x	x	x	x	o	o	o
L6	x	x	x	o	o	o	x	x	x	o	o	o	x	x	x	x	x	x	o	o	o
L7	/	/	/	/	/	/	/	/	/	/	/	/	/	/	/	/	/	/	/	/	/
L8	x	x	x	o	o	o	x	x	x	o	o	o	x	x	x	x	x	x	o	o	o
L9	x	x	x	o	o	o	x	x	x	o	o	o	x	x	x	x	x	x	o	o	o
L10	x	x	x	o	o	o	x	x	x	o	o	o	x	x	x	x	x	x	o	o	o
L11	x	x	x	o	o	o	x	x	x	x	x	x	x	x	x	x	x	x	o	o	o
L12	x	x	x	o	o	o	x	x	x	x	x	x	x	x	x	x	x	x	o	o	o
L13	x	x	x	o	o	o	x	x	x	x	x	x	x	x	x	x	x	x	o	o	o
L14	x	x	x	o	o	o	x	x	x	x	x	x	x	x	x	x	x	x	o	o	o
L15	x	x	x	o	o	o	x	x	x	x	x	x	x	x	x	x	x	x	o	o	o
L16	x	x	x	o	o	o	x	x	x	x	x	x	x	x	x	x	x	x	o	o	o
L17	x	x	x	o	o	o	x	x	x	x	x	x	x	x	x	x	x	x	o	o	o
L18	o	o	o	o	o	o	o	o	o	o	o	o	x	x	x	o	o	o	o	o	o
L19	x	x	x	o	o	o	o	o	o	o	o	o	x	x	x	o	o	o	o	o	o
L20	/	/	/	/	/	/	/	/	/	/	/	/	/	/	/	/	/	/	/	/	/
L21	x	x	x	o	o	o	o	o	o	o	o	o	x	x	x	o	o	o	o	o	o
L22	x	x	x	o	o	o	o	o	o	o	o	o	x	x	x	o	o	o	o	o	o
L23	/	/	/	/	/	/	/	/	/	/	/	/	/	/	/	/	/	/	/	/	/
L24	x	x	x	o	o	o	o	o	o	o	o	o	x	x	x	o	o	o	o	o	o
L25	x	x	x	o	o	o	o	o	o	o	o	o	x	x	x	o	o	o	o	o	o
L26	x	x	x	o	o	o	x	x	x	x	x	x	x	x	x	x	x	x	o	o	o
L27	x	x	x	o	o	o	x	x	x	x	x	x	x	x	x	x	x	x	o	o	o
L28	x	x	x	o	o	o	x	x	x	x	x	x	x	x	x	x	x	x	o	o	o
L29	x	x	x	o	o	o	o	o	o	o	o	o	x	x	x	x	x	x	o	o	o
L30	x	x	x	o	o	o	o	o	o	o	o	o	x	x	x	x	x	x	o	o	o
L31	x	x	x	o	o	o	o	o	o	o	o	o	x	x	x	x	x	x	o	o	o
L32	x	x	x	o	o	o	x	x	x	o	o	o	x	x	x	x	x	x	o	o	o
L33	x	x	x	o	o	o	x	x	x	o	o	o	x	x	x	x	x	x	o	o	o
L34	x	x	x	o	o	o	x	x	x	o	o	o	x	x	x	x	x	x	o	o	o
L35	x	x	x	o	o	o	x	x	x	o	o	o	x	x	x	x	x	x	o	o	o
L36	x	x	x	o	o	o	x	x	x	o	o	o	x	x	x	x	x	x	o	o	o
L37	/	/	/	/	/	/	/	/	/	/	/	/	/	/	/	/	/	/	/	/	/
L38	x	x	x	o	o	o	x	x	x	o	o	o	x	x	x	x	x	x	o	o	o
L39	x	x	x	o	o	o	x	x	x	o	o	o	x	x	x	x	x	x	o	o	o
L40	x	x	x	o	o	o	x	x	x	o	o	o	x	x	x	x	x	x	o	o	o

## Data Availability

The data presented in this study are openly available in [Zenodo] at [https://zenodo.org/records/21931054] (accessed on 3 September 2026).

## Supplementary Materials

The following are available online at https://zenodo.org/records/21931054 (accessed on 3 September 2026).

## Abbreviations

The following abbreviations are used in this manuscript:

1,2-DCE	1,2-Dichloroethane
AA-EQS	Annual Average Environmental Quality Standards
AOAC	AOAC International (Association of Official Agricultural Chemists)
BTEX	Benzene, Toluene, Ethylbenzene, Xylenes
CB	Chlorobenzene
DTD	Danube–Tisa–Danube (Hydro-system/Irrigation Canal Network)
EI	Electron Impact (Ionization)
EQS	Environmental Quality Standards
EU	European Union
GC	Gas chromatography
GC-MS	Gas chromatograph—Mass spectrometry
HPLC	High-performance liquid chromatography
HS-GC-MS	Headspace Gas Chromatography—Mass Spectrometry
HS-SPME	Headspace Solid-Phase microextraction
MAC-EQS	Maximum Allowable Concentrations Environmental Quality Standards
MIMS	Membrane Inlet Mass Spectrometry
MSD	Mass Selective Detector
*m*/*z*	Mass-to-Charge Ratio
NIST	National Institute of Standards and Technology
P&T	Purge-and-Trap
PCE	Tetrachloroethylene
ppb	Part Per Billion (µg/L)
RSD	Relative Standard Deviation
S/N	Signal-to-noise
Stddev	Standard Deviation
SIM	Selected Ion Monitoring
TCE	Trichloroethylene
US EPA	United States Environmental Protection Agency
VOC	Volatile Organic Compound
